# Glycemic variability and mortality in oncologic intensive care units

**DOI:** 10.1590/0034-7167-2022-0812

**Published:** 2023-10-09

**Authors:** Aretha Pereira de Oliveira, Mariana da Silva Castro, Dalmo Valério Machado de Lima

**Affiliations:** I Universidade Federal Fluminense. Niterói, Rio de Janeiro, Brazil; II Universidade Federal do Estado do Rio de Janeiro. Rio de Janeiro, Rio de Janeiro, Brazil

**Keywords:** Intensive Care Units, Blood Glucose, Mortality, Neoplasms, Glycemic Control., Unidades de Terapia Intensiva, Glicemia, Mortalidade, Neoplasias, Controle Glicêmico., Unidades de Cuidados Intensivos, Glucemia, Mortalidad, Neoplasias, Control Glucémico.

## Abstract

**Objective:**

This study aimed to investigate the association between glycemic variability and mortality in patients admitted to oncologic intensive care units.

**Methods:**

A retrospective cohort study was conducted using a convenience sample of 30 medical records of patients over 18 years of age of both sexes. Glycemic variability was measured using the standard deviation and general amplitude. Statistical analysis was performed using the receiver operating characteristic (ROC) curve and the area under the curve (AUC). The significance level (α) was set at 5% with a confidence interval (CI) of 95%.

**Results:**

The study included 14 male patients (46.67%) with a mean age of 60±15 years. A total of 1503 blood glucose samples were collected. The AUC analysis for the standard deviation did not show a statistically significant result (p=.966; 95% CI=[0.283, 0.726]). In contrast, the general amplitude had a statistically significant association with mortality (p=.049; 95% CI=[0.514, 0.916]).

**Conclusions:**

This study found that glycemic variability measured by the general amplitude was significantly associated with patient mortality in oncologic intensive care units. These findings suggest that monitoring glycemic variability may be an important factor in the management of critically ill patients in oncologic intensive care units.

## RESUMO

**Objetivo:**

investigar a associação entre a variabilidade glicêmica e a mortalidade em pacientes internados em unidades de terapia intensiva oncológicas.

**Métodos:**

estudo de coorte retrospectivo foi conduzido utilizando uma amostra de conveniência de 30 prontuários médicos de pacientes com mais de 18 anos de ambos os sexos. A variabilidade glicêmica foi medida utilizando o desvio padrão e a amplitude geral. A análise estatística foi realizada utilizando a curva ROC (*receiver operating characteristic*) e a área sob a curva (AUC). O nível de significância (α) foi estabelecido em 5% com um intervalo de confiança (IC) de 95%.

**Resultados:**

o estudo incluiu 14 pacientes do sexo masculino (46,67%) com idade média de 60±15 anos. Um total de 1503 amostras de glicemia foram coletadas. A análise AUC para o desvio padrão não mostrou resultado estatisticamente significativo (p = 0,966; IC 95% = [0,283, 0,726]). Em contraste, a amplitude geral teve uma associação estatisticamente significativa com a mortalidade (p = 0,049; IC 95% = [0,514, 0,916]).

**Conclusões:**

Este estudo encontrou que a variabilidade glicêmica medida pela amplitude geral está significativamente associada à mortalidade do paciente em unidades de terapia intensiva oncológicas. Esses achados sugerem que o monitoramento da variabilidade glicêmica pode ser um fator importante no manejo de pacientes criticamente doentes em unidades de terapia intensiva oncológicas.

## RESUMEN

**Objetivo:**

investigar la asociación entre la variabilidad glucémica y la mortalidad en pacientes ingresados en unidades de cuidados intensivos oncológicos.

**Métodos:**

Se llevó a cabo un estudio de cohorte retrospectivo utilizando una muestra de conveniencia de 30 historias clínicas de pacientes de ambos sexos mayores de 18 años. La variabilidad glucémica se midió utilizando la desviación estándar y la amplitud general. El análisis estadístico se realizó utilizando la curva ROC (receiver operating characteristic) y el área bajo la curva (AUC). El nivel de significancia (α) se estableció en un 5% con un intervalo de confianza (IC) del 95%.

**Resultados:**

El estudio incluyó a 14 pacientes masculinos (46,67%) con una edad media de 60±15 años. Se recopilaron un total de 1503 muestras de glucemia. El análisis AUC para la desviación estándar no mostró ningún resultado estadísticamente significativo (p = 0,966; IC del 95% = [0,283, 0,726]). Por el contrario, la amplitud general tuvo una asociación estadísticamente significativa con la mortalidad (p = 0,049; IC del 95% = [0,514, 0,916]).

**Conclusiones:**

Este estudio encontró que la variabilidad glucémica medida por la amplitud general está significativamente asociada con la mortalidad del paciente en unidades de cuidados intensivos oncológicos. Estos hallazgos sugieren que el monitoreo de la variabilidad glucémica puede ser un factor importante en el manejo de pacientes críticamente enfermos en unidades de cuidados intensivos oncológicos.

## INTRODUCTION

Hyperglycemia is a common occurrence during critical illness, affecting patients with and without diabetes. It has been associated with oxi-hemodynamic, metabolic, and nutritional alterations resulting from the acute clinical state and ICU treatment, and is strongly associated with increased mortality^(1-4)^.

Since the early 2000s, glycemic control in the ICU has been an important topic of discussion, with studies on clinical and surgical patients demonstrating decreased mortality rates in patients with blood glucose maintained between 80 and 110 mg/dL^(5-6)^. However, the largest multicentric clinical trial conducted in 42 ICUs did not confirm these findings, with patients maintained normoglycemic experiencing a higher incidence of hypoglycemia and increased mortality, suggesting that it is safe to tolerate a blood glucose level up to 180 mg/dL ^(7)^.

Based on the suggestion that routine procedures performed on ICU patients could increase resting energy expenditure ^(8)^, a recent uncontrolled clinical trial conducted with 30 cancer patients demonstrated that bed baths performed with wet wipes reduced mean glycemia by 7.01% ^(9)^.

Recently, other parameters such as hypoglycemia occurrence, glycemic gap, and glycemic variability during ICU stay have been identified as valuable prognostic markers, particularly for mortality ^(10-11)^. Glycemic variability can be a confounding factor in studies that only analyze target blood glucose values ^(12)^.

Glycemic variability refers to blood glucose fluctuations over time, which can be evaluated throughout the day or during specific hospitalization periods. These fluctuations are not reflected in the mean glycemia value, and some authors are suggesting its use as an additional measure in glycemic control ^(13)^.

In addition to the metabolic peculiarities of cancer’s evolution and treatment, studies related to glycemic evaluation of cancer patients are rare, and this population is often included in non-specific analyses in general ICU. This study aims to test the hypothesis that glycemic variability during the ICU stay of cancer patients increases mortality.

## OBJECTIVE

To test the association between glycemic variability and mortality.

## METHODS

### Ethical considerations

This study was conducted in accordance with the principles of the Declaration of Helsinki and approved by the Research Ethics Committee of the institution under opinion number 1.911.346. Patient consent was waived since this was a retrospective study that utilized only data from medical records.

### Study design

This was a retrospective cohort study using a quantitative approach. It was conducted in the Intensive Care Unit (ICU) or Post-Operative Unit (POU) of a leading health institute in cancer treatment in Latin America, between October and December 2016, in accordance with the STROBE guidelines.

### Sample, inclusion, and exclusion criteria

Non-probabilistic convenience sample composed of 30 medical records of patients consecutively recruited using the following eligibility criteria: both sexes, over 18 years of age, admitted to the Intensive Care Unit (ICU) or Post-Operative Unit (POU). Follow-up time was 28 days from admission.

Patients with a length of stay in intensive units of less than 24 h, with a definition of end-of-life care recorded in the medical record, with ongoing hospitalization in intensive units were excluded. In cases of readmission, only the first admission was considered in the analyses. The exclusion of patients defined as end-of-life care was done as a way of controlling bias, given that the inexorable outcome of these individuals is death, which could be a confounding factor in the analysis.

### Study protocol

Eligible patients were preliminarily selected by evaluating records in the Admission Books of the study units. A total of 52 patients who could be included in the study were identified. Subsequently, the medical records of all patients consecutively admitted to the clinical and surgical ICU of an oncology hospital located in the city of Rio de Janeiro, Brazil, during the months immediately preceding the approval of the project by the Ethics Committee in Research of the institution, were reviewed.

Demographic and clinical data, including gender, age, reason for ICU admission, and history of diabetes, in addition to blood glucose measurements recorded, were collected during the three-month data collection period. Patients were followed up until discharge or death from any cause in the ICU.

All blood glucose measurements were performed by evaluating capillary blood obtained by perforating the skin of a finger with a lancet or arterial blood obtained through an invasive pressure catheter and recorded on the patients’ water balance sheets. The patient was considered hyperglycemic when capillary blood glucose was above 180 mg/dL, hypoglycemic when blood glucose was below 70 mg/dL, and severely hypoglycemic in cases of blood glucose below 40 mg/dL, according to the values established in the NICE-SUGAR^(7)^.

Glycemic variability was calculated using both the standard deviation and the amplitude (difference between maximum and minimum values) during hospitalization, according to a study published in 2019^(14)^.

### Data analysis

The collected information was entered into an electronic spreadsheet and subjected to analysis procedures using the statistical package SPSS PASW 17.0 for Windows, owned by IBM. Data entry was performed by two independent researchers and later compared to minimize typing errors.

Descriptive statistics were performed using measures of central tendency, represented by the mean and median, and measures of dispersion, represented by the standard deviation (SD). After analyzing the normality of data distribution using the Shapiro-Wilk test, inferential statistics were performed by analyzing the Receiver Operating Characteristic (ROC) curve and the area under the curve. A significance level (α) of 5% and a Confidence Interval of 95% were previously established.

## RESULTS

**Figure 1:**
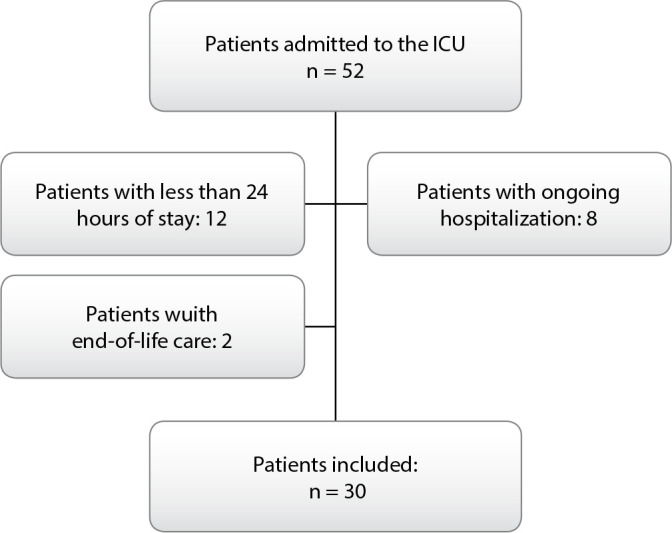
Eligible patients recruitment flow diagram, Rio de Janeiro, Rio de Janeiro, Brazil, 2017

During the data collection period, 52 patients were admitted to the study units, but 22 were excluded. Two of these patients received end-of-life care, eight were still hospitalized in the study units, and 12 had a shorter length of stay in the intensive units that was less than 24 hours, resulting in a sample of 30 medical records as depicted in Figure 1.

The sample comprised 30 medical records of cancer patients, with 14 (46.67%) being male and a mean age of 60±15 years. A total of 1503 blood glucose samples were collected, with an average of 50 assessments per patient. Among the patients, 10 (33.3%) had no hyperglycemic peak during hospitalization, and of these, three died. In contrast, eight (26.67%) experienced at least one episode of hypoglycemia, and six of them died. The average general blood glucose range for the sample was 123±65 mg/dL, and the length of stay ranged from two to 28 days, with an average of 12±9 days. The main demographic, clinical, and mortality characteristics according to the occurrence of hyperglycemia are summarized in Table 1.

**Table 1:** Demographic, clinical, glycemic and mortality profile of patients in clinical and surgical ICU of a cancer hospital, Rio de Janeiro, Rio de Janeiro, Brazil, 2017

Variable	Hyperglycemia during ICU stay				*p* value	95% CI
	Yes		No			
	Mean (SD)		Mean (SD)			
Age^1^	64.50(10.57)		50.30(17.86)		.010	[-24.796; -3.624]
Higher Glycemia^2^	235.60(44.40)		142.80(22.03)		< .001	[-117.645; -67.955]
Lowest Glycemia^1^	79.20(18.70)		74.80(16.67)		0.535	[-18.734; 9.934]
General amplitude of glycemia^2^	156.40(55.25)		68.00(32.07)		< .001	[-121.185; -55.615]
	**n**	**%**	**n**	**%**		
Sex^3^						
Female	10	33.33	6	20.00	.709	[.138; .612]
Male	10	33.33	4	1.34		[.049; .522]
Death in ICU^3^						
Yes	10	33.33	3	10.00	.440	[.001; .460]
No	10	33.33	7	2.34		[.178; .646]

1 T Test for homoscedastic independent samples;

2 T Test for heteroskedastic independent samples;

3 Fisher’s Exact Test.

Table 1 analysis revealed that the mean age of patients who experienced at least one episode of hyperglycemia during their ICU stay was significantly higher than those who did not (p = .010), as well as the glycemic amplitude (p < .001). There were no significant differences in gender and ICU mortality (p > .05). The mean length of ICU stays among patients who had hyperglycemia was significantly longer (14.60 days) than those who did not (7.20 days), as assessed using the T-test for homoscedastic independent samples (p = .037).

Regarding the eight patients who had hypoglycemia, the mean length of stay was 16 days, while those who did not have it remained hospitalized for an average of 11 days (p = .174), and six of them died during their stay in the ICU (p = .035). Two patients experienced severe hypoglycemia (< 40 mg/dL), and both died.

**Figure 2:**
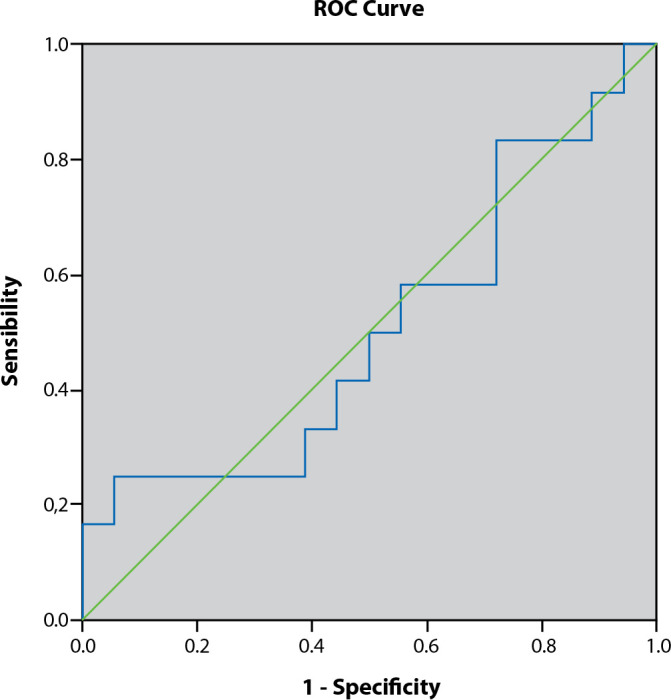
ROC curve to determine the sensitivity of the standard deviation as a measure of glycemic variability to predict mortality in cancer patients in the ICU, Rio de Janeiro, Rio de Janeiro, Brazil, 2017

**Figure 3:**
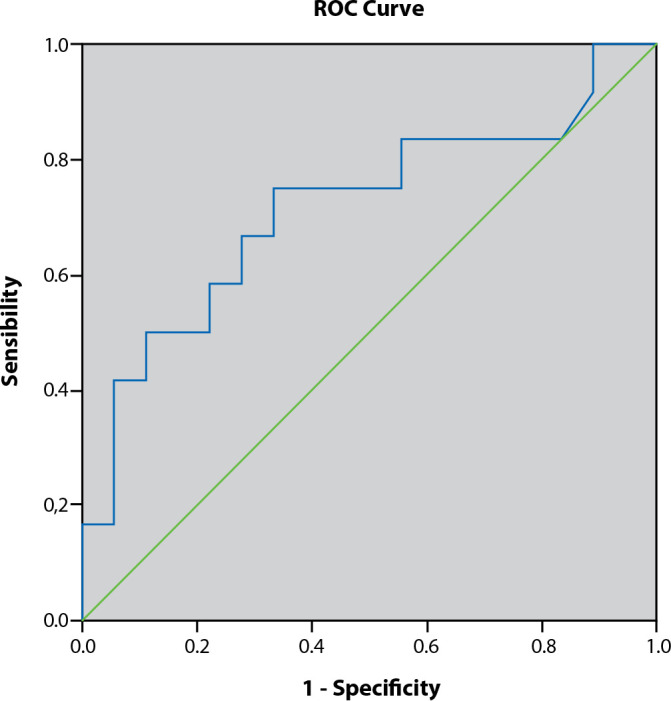
ROC curve to determine the sensitivity of general amplitude as a measure of glycemic variability to predict mortality in cancer patients in the ICU, Rio de Janeiro, Rio de Janeiro, Brazil, 2017

The ROC curve and the area under the curve were analyzed to identify differences in the prediction of blood glucose variability by the standard deviation (Figure 2) and the general amplitude (Figure 3) on the 28-day mortality of cancer patients in intensive care units.

The analysis of the area under the ROC curve for the standard deviation did not yield a statistically significant result (p = .966; 95% CI = [.283; .726]), unlike the analysis of the general amplitude (Figure 3), which had a p-value of .049 and 95% CI = [.514; .916].

These results demonstrate that, in the sample studied, the glycemic variability calculated by the general amplitude was a better predictor of mortality in a univariate analysis than the variability calculated by the standard deviation. Regarding other variables, the results showed no difference in the occurrence of hyperglycemia between genders (p = .709), the presence of diabetes (p = .451), or between the reasons for hospitalization (p = .707). Similarly, there was no difference in the occurrence of hypoglycemia between genders (p = .463), the presence of diabetes (p = .271), or between medical or surgical hospitalization (p = 1.000).

## DISCUSSION

In this retrospective study, conducted with critically ill patients with oncological diseases who were hospitalized in intensive care units, including individuals with and without a history of diabetes, the glycemic parameters analyzed were associated with adverse outcomes, such as death and longer ICU stays. The occurrence of hypoglycemia and glycemic variability calculated by the general range were significantly associated with 28-day mortality.

It should be noted, however, that there is a lack of evidence regarding the best blood sample (capillary, arterial, or venous) to analyze glycemia using hemoglucotest devices in intensive care units. In this study, both capillary and arterial samples were used, without being possible to identify them through the records.

Although hyperglycemia is a common metabolic occurrence in critically ill patients ^(5-6,15)^, the ideal glycemic parameters remain unknown, and the discussion is still controversial. In this sense, we highlight the early interruption of a multicenter clinical trial that sought to assess the reduction in mortality in the ICU with the implementation of strict control in 2009, due to the high incidence of severe hypoglycemia in this group. The analyzed results showed no difference in the mortality rate in the ICU, the length of stay in the ICU, and the length of hospital stay between the two groups^(16)^.

Another important discussion refers to the definition of which glycemic analysis parameter would be more determinant in the occurrence of negative outcomes. Several studies presented analyses regarding the incidence of hyperglycemia^(5-7,14)^ and hypoglycemia^(17-19)^. The incidence of hyperglycemia in the first 24 hours of ICU admission has also been identified as a predictor of mortality in different populations^(20-22)^. More recently, assessments of glycemic variability and the ratio of hyperglycemia due to stress^(23)^ have been discussed and associated with outcomes such as shock, the need for renal replacement therapy, and mechanical ventilation^(14)^.

Glycemic variability, despite being discussed as a better predictor of mortality in severe disease^(14,24-25)^, still presents different methods of quantification, such as standard deviation, changes in absolute mean blood glucose per hour, and glycemic lability index^(13,26)^, with no consensus on the best way to calculate, which can cause significant differences in the results presented, making it impossible to compare them in different populations.

This study used the standard deviation and the glycemic amplitude presented by the patients as forms of quantification of variability, in line with national and international studies, highlighting the significant difference in the mean variability presented in our study in the two forms of calculation.

It is notable that our sample was composed exclusively of patients undergoing treatment for oncological diseases, with peculiarities that need to be considered. Some evidence suggests a significant role for hyperglycemia in all oncological disease processes, from oncogenesis to treatment^(27-28)^. Furthermore, hyperglycemia increases the risk of cancer and contributes to its progression and mortality. Several types of cancer advance more aggressively under hyperglycemic conditions, particularly tumors of the liver, pancreas, breast, and endometrium^(29-30)^. In a meta-analysis of eight studies comprising 4,342 patients, hyperglycemia was associated with lower disease-free survival and lower overall survival^(31)^. Although studies claim that hyperglycemia increases the risk of developing cancer, affects progression and mortality, evidence about the possible metabolism and molecular events responsible for these changes is scarce.

It should be noted that there were no differences between the occurrence of hyperglycemia in patients with and without diabetes, in line with other studies^(4-6,9)^. Glycemic alterations in critically ill patients are mainly related to metabolic stress, the release of stress hormones, drug use, and inflammatory or infectious processes.

### Limitations

As this was a retrospective study, the data were dependent on the records maintained by the nursing teams, which varied in the amount and frequency of measurements taken for each patient. Additionally, since blood glucose measurements are intermittent, the lack of equipment that enables continuous monitoring may lead to the loss of critical information. The diabetes history recorded at the institution relied solely on patients’ self-declaration and lacked confirmation by laboratory tests or disease control assessment.

### Contributions to the Area

Our findings indicate a potential link between glycemic variability and mortality, which could have a significant impact on the work of critical care nurses. The nursing team must develop tailored strategies and protocols for managing hyperglycemia in this population. It may be necessary to avoid changing ongoing glucose control, and patients with cancer and hyperglycemia must be monitored more closely than others.

## CONCLUSION

Our study revealed that glycemic variability, as measured by general amplitude, and the occurrence of at least one episode of hypoglycemia during hospitalization in oncology Intensive Care Units, were significantly associated with patient mortality. Of particular note is the high absolute mortality among hypoglycemic patients. These results suggest that glycemic parameters can have a significant impact on the outcomes of cancer patients and should be closely monitored and controlled by the nursing team. We found no significant relationships between glycemic variables and sex, reason for ICU admission, or history of diabetes in the studied sample.

## Data Availability

*https://doi.org/10.48331/scielodata.0D3MFA*
